# Descriptive cross sectional study on prevalence, perceptions, predisposing factors and health seeking behaviour of women with stress urinary incontinence

**DOI:** 10.1186/1472-6874-14-78

**Published:** 2014-07-02

**Authors:** Jennifer Perera, Dinoo S Kirthinanda, Sujani Wijeratne, Thanuja K Wickramarachchi

**Affiliations:** 1 Department of Microbiology, Faculty of Medicine, University of Colombo, P O Box 271, Colombo, Sri Lanka

**Keywords:** Stress urinary incontinence, Prevalence, Risk factors, Health seeking behaviour

## Abstract

**Background:**

Stress urinary incontinence (SUI) leads to considerable physical and psychological morbidity. The highest prevalence reported was found in Caucasian Americans (range 23% -67%) and the lowest in Singaporean females (4.8%). The study assessed the prevalence, perceptions, predisposing factors and health seeking behaviour of women with SUI in an Asian setting which may have different sociocultural implications.

**Methods:**

400 consecutive women >20 years of age attending the outpatient department of a tertiary care hospital in Sri Lanka, for non-urinary conditions were studied over a 3 week period using an interviewer administered questionnaire. SUI was diagnosed on clinical history alone when leakage of urine occurred either with coughing, sneezing, walking or lifting heavy objects. The severity was graded using the Finnish Gynaecological Society’s Urinary Incontinence Severity Score (UISS). Data were analysed using SPSS version 20. Odds ratios were calculated using univariate and multivariate analysis.

**Results:**

Ninety three (23.33%) had SUI and only 12 (12.9%) had sought treatment. The prevalence among women >50 years of age was 34.71% ( n = 121) compared to 18.28% (n = 279) in those ≤50 years. 25 (26.88%) had mild SUI, 66 (70.97%) moderate and 2 (2.15%) severe as per UISS. SUI was perceived as an illness by 210 (52.5%). SUI was significantly associated with pregnancy, parity, vaginal delivery, complicated labour, diabetes mellitus, chronic cough, constipation and faecal incontinence (p < 0.05).

Among those affected main reasons for not seeking medical advice included; being embarrassed (n = 27, 33.33%), not knowing that it is remediable (n = 23, 28.40%), perceiving SUI to be a normal consequence of childbirth (n = 19, 23.46%) and having to attend to needs of the family (n = 12, 14.81%). None who had been pregnant (n = 313) had received advice on postnatal pelvic floor exercises. SUI interfered with social activities (71;76.34%), sexual function (21; 22.58%) and resulted in despair (67; 72.09%). It was associated with clinically diagnosed candidiasis (50; 53.76%) and soreness in the perineal region (49; 52.69%).

**Conclusions:**

SUI is a common and neglected gynaecological problem with poor healthcare seeking behaviour. Community based education may help to minimize the occurrence and improve the quality of life of those affected.

## Background

Stress urinary incontinence (SUI) occurs when bladder pressure exceeds urethral closure pressure, causing transient sphincter opening and urine loss. It is caused by physical activities such as walking, lifting heavy objects, coughing, sneezing or any other activity that creates a sudden increase in intra-abdominal pressure [1]. The International Incontinence Society has standardized the definition of urinary incontinence as “the complaint of any involuntary leakage of urine,” and suggests inclusion of the type, frequency, severity, impact on the quality of life etc. as additional descriptive criteria [2]. While SUI is more common in older females, a significant proportion of young and middle-aged women also experience SUI [3]. Between 5% to 10% of those with SUI may have severe adverse physical, psychological and social effects [4,5].

The highest prevalence estimates were found in Caucasian Americans and ranged from 23% [6] to 67% [7]*.* Among the lowest were Singaporean females, in whom the prevalence was 4.8% [8]. Although the prevalence of SUI is high, the health seeking behaviour was found to be low worldwide, with only 13%–55% of women with symptoms of incontinence seeking medical care [9]. Many do not regard their symptoms as abnormal or serious [9-11] and some even hope that it may improve with time [12]. Older women tend to regard incontinence as a normal consequence of aging, and health-care providers may reinforce this belief [13]. Embarrassment prevents some from consulting a doctor and others believe that SUI cannot be treated [9,14] or fear that surgery is the only available treatment option [12]. In a community-based descriptive cross-sectional study done to explore barriers to healthcare, fear of vaginal examination, shame and embarrassment, and belief that SUI was a natural consequence of aging and childbirth were reasons for not seeking care [15].

Both young as well as old women tend to conceal urinary incontinence in their daily lives, by keeping the bladder empty, limiting social interactions and using hygiene measures [16]. Although urinary incontinence does not appear to increase mortality, the medical morbidity is substantial leading to local candida infection, cellulitis, pressure sores, constant skin irritation and sleep deprivation due to nocturia [17]. Psychological morbidity includes poor self-esteem, social withdrawal, depression, sexual dysfunction due to embarrassment, and curtailed social and recreational activities [17]. Stigma leading to social isolation and internalised shame negatively correlated with quality of life of patients suffering from SUI [18].

Multiple risk factors have been identified as responsible for development of SUI. The prevalence of UI increases with age. Hormone therapy, hysterectomy, parity, having a BMI over 30 kg/m^2^, smoking, having diabetes and being physically or sexually active, tended to increase the risk of UI [14,19-21]. The overall prevalence of incontinence increased significantly with long standing cough [22] and was associated with a high intake of caffeine [23]. Caffeine, in addition to having a diuretic effect may also affect smooth muscle contractions [23].

The impact of SUI on the quality-of-life may be used to guide the intensity of treatment [24]. The Finnish Gynaecological Society’s urogynaecological working group has designed Urinary Incontinence Severity Score questionnaire (UISS), which has been widely used in clinical practice, to assess severity of symptoms and its impact on daily life [25]. The current treatment pathway for SUI is initial conservative therapy, including dietary advice, weight reduction, management of bowel problems and pelvic floor exercises followed by referral to secondary care with surgical intervention if conservative treatment fails to sufficiently relieve the symptoms. A study in primary care reported that only 13% of women seeking help were referred to secondary care suggesting that primary care has a significant role to play in addressing this unmet need in the community [26].

The information available on SUI from non-Western cultures is sparse. Hence, a study on SUI from an Asian country would add value to the available information as this topic has social and cultural implications. Additionally the information may be useful in formulating policies, health education programmes and treatment guidelines specific for the population assessed. Thus the objective of this study was to determine the prevalence, degree of severity, identify associated factors and study the perceptions and health seeking behaviour of women with SUI attending a health care facility.

## Methods

Ethical clearance for the study was obtained from the Ethics Review Committee of the Faculty of Medicine, University of Colombo, Sri Lanka (Ref No –EC/08/010). Sample size required for the study was calculated by using the formula n = t^2^xp(1-p)/m^2^ (**n** = required sample size, **t =** confidence level at 95% (standard value of 1.96), **p =** estimated prevalence of UI in the project area, **m =** margin of error at 5% (standard value of 0.05) applying 50% probability for prevalence due to lack of information on prevalence of SUI in Sri Lanka. A non-interventional descriptive cross sectional study was carried out over three week period on 400 consequent women >20 years of age attending the outpatient department of a tertiary care hospital in Sri Lanka. Informed written consent was obtained from study participants prior to conducting the study and the questionnaire was administered by a female interviewer in a setting that provided privacy during the interview.

A pre-tested, expert validated (5 content experts were used in the validation of content), questionnaire was used as the study tool. Content validity of the questionnaire was established as each question scored more than an average of 3 in the scale used for validation which ranged from 0–5. The questionnaire was used to gather information on prevalence, knowledge, perceptions, health seeking behaviour and associated factors that may predispose to urinary incontinence. SUI was diagnosed if the patient confirmed the presence of any involuntary leakage of urine with coughing, sneezing, walking or lifting heavy objects etc. on direct questioning. Gynaecological examination was not performed to confirm the findings. The severity of incontinence was graded using the Finnish Gynaecological Society’s Urinary Incontinence Severity Score questionnaire (UISS) [22]. [Additional file 1: UISS]. Data were analysed using SPSS version 20. Chi square test was performed and odds ratios were calculated in the univariate analysis. Multivariate analysis was carried out using variables which were significantly (p ≤ 0.05) associated with UI in the univariate analysis.

## Results

The mean age of the study population was 41.94 years with a range of 21–88 years. 93 (23.3%) admitted having SUI and among them 54 (58.06%) were having symptoms of SUI for over a year and a similar number had at least daily episodes of incontinence (Table 1). Among the incontinent women only 12 (13%) had sought treatment. The treatment seeking behaviour was related to the degree of severity of UI (Table 1).

**Table 1:** Frequency of urinary incontinence and treatment seeking behaviour (n = 93)

**Frequency**	**Number of patients (%)**	**On treatment (% of the affected category)**
No incontinence	307 (76.75)	-
Occasionally	21 (5.25)	1 (4.76)
At least once a month	12 (3)	1 (8.33)
At least once a week	6 (1.5)	1 (16.6)
At least once a day	44 (11)	5 (11.4)
More than once daily	10 (2.5)	4 (40%)

**Figure 1:**
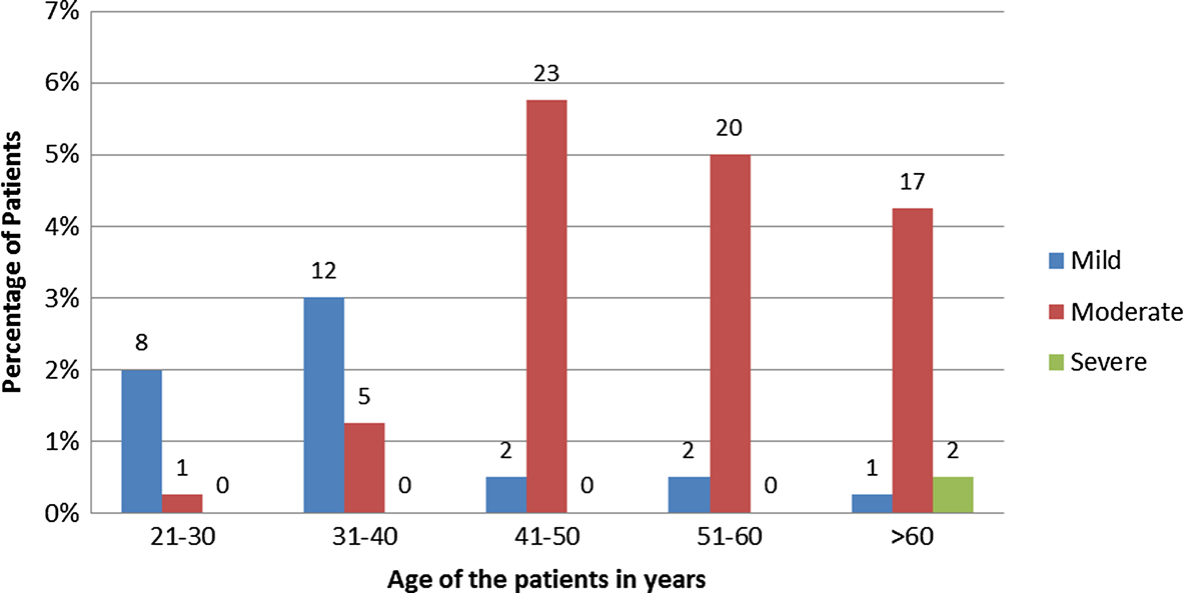
Prevalence and severity of SUI in different age groups.

The affected group provided a variety of reasons for not seeking treatment as summarised in Table 2. The awareness of the study population on possible healthcare interventions for SUI was quite low and many perceived it to be a common consequence of child bearing or aging. Nearly half of the study population (n = 190, 47.50%) did not perceive SUI as an illness and more women without SUI viewed this as a normal phenomenon (55.7%) compared to those affected (23.46%).Twenty five patients (26.88%) had mild urinary incontinence, in 66 (70.97%) it was moderate and 2 (2.15%) had severe SUI when classified according to the urinary incontinence severity scale. As expected, both the prevalence and the severity of incontinence increased with advancing age as shown in Figure 1. However a significant number of women in the middle and young age groups complained of SUI (Figure 1).

**Table 2:** Main reasons for not seeking treatment (n = 81)

**Main reason for not seeking treatment**	**Number (%)**
Thinking that UI is normal with ageing and parity	19 (23.46)
Feeling embarrassed to consult a doctor	27 (33.33)
Not knowing that treatment is possible or thinking surgery as the only available treatment option	23 (28.40)
Being busy with other priorities of the family	12 (14.81)

In the study population multiparity was identified as a significant risk factor for SUI compared to uniparity or nulliparity. Miscarriages were taken into consideration excluding the first trimester miscarriages. Complicated labour was associated with UI significantly more, compared to uncomplicated labour. Women who had vaginal deliveries were significantly affected by UI in contrast to women who had undergone a lower segment Caesarean section (LSCS). Diabetes mellitus, chronic cough, faecal incontinence (elicited by verbal inquiry) and constipation showed a significant association with UI. Abdominal surgery other than LSCS, bronchial asthma and regular consumption of coffee did not seem to increase the prevalence of urinary incontinence significantly (Table 3).

**Table 3:** Risk factors for urinary incontinence

**Risk factors (reference)**		**No. with UI (%)**	**No. without UI (%)**	**Unadjusted OR**	p value*
Past pregnancy [9]	None	85 (27.2)	228 (72.8)	3.68	<0.001
	One or more	8 (9.2)	79 (90.8)		
Parity [9]	Nulliparity or uniparity	22 (13.8)	13 (86.2)	2.63	<0.001
	Two or more	71(29.6)	169 (70.4)		
Normal vaginal delivery [19]	No	80 (27.7)	209 (72.3)	2.89	0.001
	Yes	13 (11.7)	98 (88.3)		
Abdominal surgery [19]	No	67 (22.1)	236 (77.9)	1.29	0.341
	Yes	26 (26.8)	71 (73.2)		
Bronchial asthma [20]	No	67 (21.5)	245 (78.5)	1.53	0.113
	Yes	26 (29.5)	62 (70.5)		
Prolonged cough [22]	No	64 (19.2)	269 (80.8)	3.21	<0.001
	Yes	29 (43.3)	38 (56.7)		
Constipation [19]	No	81 (22.0)	288 (88.0)	2.25	0.034
	Yes	12 (38.7)	19 (61.3)		
Menopause [9]	No	75 (23.9)	239 (76.1)	0.844	0.565
	Yes	18 (20.9)	68 (79.1)		
Faecal incontinence	No	88 (22.4)	304 (77.6)	5.76	0.026**
	Yes	05 (62.5)	03 (37.5)		
Regular coffee consumption [23]	Yes	12 (25.7)	33 (74.3)	1.23	0.565
	No	81(22.8)	274 (77.2)		
Diabetes mellitus [19]	No	70 (20.2)	276 (79.8)	2.92	<0.001
	Yes	23 (42.6)	31 (57.4)		

*p value was calculated with Chi-square test.

**Continuity correction of small values applied.

Table 4 shows the adjusted odds ratios of risk factors of SUI with logistic regression analysis. The number of children, undergoing vaginal delivery, having chronic cough, having constipation, having faecal incontinence and presence of diabetes mellitus were included as independent variables as these factors showed a significant association (p < 0.05) in the initial univariate analysis. The variable ‘past pregnancy’ was not included in the model as it was highly correlated with the variable ‘number of children’. As shown in Table 4 when adjusted for the effect of variables retained in the final model, having two or more children, chronic cough, constipation and diabetes mellitus were significant predictors of SUI.

**Table 4:** Adjusted risk factors for urinary incontinence

**Risk factors**	Adjusted OR*	**(95% CI)**	**p value**
Chronic cough	2.8	(1.5–5.1)	0.001
Constipation	2.8	(1.2–6.8)	0.015
Diabetes mellitus	2.0	(1.002–3.8)	0.049
Having two or more children	2.9	(1.6–5.2)	0.000

*The variables chronic cough, constipation, diabetes mellitus, having two or more children, undergoing vaginal delivery and faecal incontinence were included in the final model used for multivariate analysis.

The familial tendency for SUI could not be assessed as none of the study participants could comment on the presence or absence of SUI among family members. As there were only a small number of patients who were smokers (n = 2) or using hormone replacement therapy (n = 3) these factors were not further analysed, and none of them belonged to the affected group. The fact that none of the mothers (n = 310) had received advice on pelvic floor strengthening exercises following child birth was a notable finding of the data analysis.

In the affected women the main sequelae that were associated with SUI were; interference with social activities and embarrassment (71, 76.34%), frustration and disappointment about life (67, 72.09%), vaginal candidiasis (50, 53.76%), perineal soreness (49, 52.69%), sleep disturbances (41, 44.09%) and interference with sexual activities (21, 22.58%) while the majority had more than one of the above mentioned complications or issues.

## Discussion

The prevalence of urinary incontinence in the study population was 23.3% and this figure is within the range (4.8 - 67%) of that in published data [6-8,27]. In spite of over half of the affected population (58.06%) has been experiencing urinary incontinence for more than one year, the treatment seeking behaviour was poor with only 12.9% of women seeking medical care. It was quite surprising that only about 16.7% of the patients who complained of daily episodes had sought treatment. These figures are similar to findings from Australia [16]. Studies from Norway and New Zealand have shown that 25%-33% women with SUI have sought medical advice [9,11] and comparatively more women from those countries appear to seek care for SUI when compared with our findings.

Similar to what has been observed in China [18], main reason for not obtaining treatment was, being embarrassed to consult a doctor (33.3%) while lack of awareness of the illness and available treatment options contributed significantly. A substantial number (23.5%) perceived that SUI to be a normal consequence of ageing and pregnancy and reflects the poor knowledge of SUI in the study group. Although pelvic floor exercises are known to strengthen the pelvic floor muscles which in turn reduce the occurrence of genital prolapse and urinary incontinence [28], none of the women in our study were aware of it. In Scotland most of the women (77.9%) were aware of the pelvic floor exercises. Their main means of obtaining information were books and mid wives [29].

The SUI in Sri Lanka is initially managed conservatively and includes dietary advice, weight reduction, management of bowel problems and pelvic floor exercises. The patients are referred to secondary care with surgical intervention if conservative treatment fails to sufficiently improve the symptoms. As many of the affected women were not aware of the preventive measures and available treatment options it would be beneficial to train community midwives on raising awareness among women. As a considerable number (14.8%) of women were busy attending to the wellbeing of their families and appear to have neglected attending to their problem due to lack of time. This indicates how women tend to prioritise between their needs and demands made of them.

Although the prevalence and severity of SUI was higher in older age groups, a significant proportion of women belonging to younger age groups too experienced SUl (Figure 1). This is consistent with available data from the Northern Europe [19]. With regard to the severity of UI, only 26.9% of patients had mild urinary incontinence and the majority (73.1%) had moderate to severe incontinence when assessed using the urinary incontinence severity scale. High percentage of moderate to severe UI together with very poor treatment seeking behaviour may be responsible for the complications experienced by the study group. SUI affected their daily life leading to interference with social activities considerably, and effects of SUI on social life were by far greater than health related morbidity.

Similar to findings from a study on women aged ≥45 years in Denmark [14], urinary incontinence was significantly associated with pregnancy and parity. Lack of optimum peri-natal and post-natal care may have contributed to the high prevalence rates of SUI observed in this study. This assumption is reinforced by the fact that vaginal delivery and complicated labour significantly increased the risk of SUI. Injury to pelvic floor muscles occurring during vaginal delivery may weaken the muscles leading to SUI in later life and provides a plausible explanation for the higher prevalence of UI with pregnancy and parity. Although abdominal surgery (other than LSCS), bronchial asthma and regular consumption of coffee did not seem to increase the prevalence of SUI in our study, these have been identified as risk factors in previous studies from the Northern Europe, Canada and United States [19,22,23]. Similar to the findings in the Canadian study [22] presence of chronic cough significantly increased the risk of SUI. This may be because the chronic cough results in frequent outbursts of high abdominal pressure which in turn lead to exhaustion of the pelvic floor muscles and other supporting mechanisms [20]. Constipation was identified as a risk factor in our study and is similar to findings from Canada [22]. A survey which included a large number of patients (n = 9340) from Norway, has shown a significant association between long standing diabetes mellitus and urinary incontinence [30] which was similar to the findings of this study. Type 2 diabetes-related microvascular damage could potentially affect the pelvic floor and lead to dysfunction of the bladder and sphincter muscles [19]. Presence or lack on any familial tendency for SUI could not be elicited from this study due to lack of information. However a large population based cross sectional study conducted in Norway has shown that the daughters of mothers with urinary incontinence has an increased risk for urinary incontinence [31].

### Limitations

The study was limited to a population that accessed a state health care service which is provided free of charge. As 50% the healthcare services provided at primary care are private and fee levying, the findings of this study cannot be generalised to the whole country. Also none of the patients were examined gynaecologically and the presence of SUI was determined by verbal inquiry alone and this may have reduced the precision of arriving at a diagnosis of SUI.

## Conclusion

A considerable proportion of the study population suffered from SUI and in a substantial proportion this affected the quality of their life. The findings of this study clearly indicate that adverse physical and psycho-social effects of SUI are substantial and require the attention of the healthcare system.

Primary prevention of SUI should be encouraged and increasing public awareness about pelvic floor exercises to increase the pelvic floor muscle strength particularly during and after pregnancy would definitely help in reducing prevalence of SUI. This should be further emphasised in curricula of relevant health professionals such as midwives. In Sri Lanka the primary health care workers (who are mostly women) work closely with the community with regard to healthcare services and health education at grass root level. It would be important to assess the knowledge, beliefs and attitudes of these healthcare staff regarding SUI as this will influence patients’ perceptions on SUI. In this regard further research is recommended to assess knowledge and attitudes of primary care health staff as this would have a direct impact on the care provided to women.

Providing adequate knowledge to women on SUI through health education would improve the knowledge on prevention and available treatment options. This may change perceptions of women on SUI leading to a better quality of life of the affected population. It is also recommended that assessment of urinary incontinence includes a description of the effect of urinary incontinence on the physical, psychological and social domains of health even in the primary level evaluation when SUI is suspected. This would enable determining the optimum type of management for an individual patient.

## Abbreviations

SUI: Stress urinary incontinence; UI: Urinary incontinence; UISS: Urinary incontinence severity score.

## Competing interests

The authors declare that they have no competing interests.

## Authors’ contributions

JP contributed by conception of the study, participation in its design, coordination of logistics and revised it critically for its intellectual content and approved the final manuscript. DSK, SAW designed the study, collected and tabulated the data, and helped in interpretation of data. TKW did statistical analysis, interpretation of data and drafted the manuscript. All authors read and approved the final manuscript.

## Authors’ information

JP is Senior Professor, Faculty of Medicine University of Colombo, Sri Lanka. Her educational qualifications are MBBS, MD (Microbiology) (Col), MBA (Wales), PgDMedEd (Dundee), PgDWomen’sStu (Col).

DS, SA and TK are pre intern medical graduates of the Faculty of Medicine, University of Colombo, Sri Lanka.

## Pre-publication history

The pre-publication history for this paper can be accessed here:

http://www.biomedcentral.com/1472-6874/14/78/prepub

## Supplementary Material

Additional file 1 **Urinary Incontinence Severity Score (UISS) [**[22]**].** Click here for file
