# Supplementary material for: Descriptive cross sectional study on prevalence, perceptions, predisposing factors and health seeking behaviour of women with stress urinary incontinence
Source: BMC Womens Health. 2014 Jul 2;14:78. doi: 10.1186/1472-6874-14-78 (PMC4094634; doi:10.1186/1472-6874-14-78)
Supplement: Additional file 1 — Urinary Incontinence Severity Score (UISS) [[22]]. [file 1472-6874-14-78-S1.docx]

**Additional file**

**Urinary Incontinence Severity Score(UISS)** [22]

**Questionnaire design**

The UISS questionnaire consists of 10 questions with a three-point scoring.The items have been derived from clinical experience and review of the literature. There are four items designed to quantify the amount of leakage and 6 items referring to the degree to which UI affects aspects of women’s daily lives including social interactions, physical activities and sexual function.

**Scoring system**

- Not at all [0]
- Sometimes [1]
- Often [2]

**Interpretation of results**

The sum of the scores is calculated. It ranges from 0-30. The sum of the score 0-5 was categorized as mild in severity, 6-15 as moderate and above 15 as severe.

The questionnaire includes items not pertinent to all patients (work, sexual life). Thus the UISS is expressed as the percentage of the patient’s score of the possible maximum produced by the questions the patient has answered.

| 1. Do you experience urine leakage not related to effort or position (for example lying down)? |
| --- |
| 2. Do you experience urine leakage related to minor physical activity (e.g. walking or rising)? |
| 3. Do you experience urine leakage related to sudden, strong physical activity or even coughing or sneezing? |
| 4. Has urine leakage disturbed your daily chores (shopping, cooking, housecleaning etc.)? |
| 5. Has urine leakage disturbed your employment (client service, work performance etc)? |
| 6. Are you afraid that others will notice your problem (fear of your odour or wetness etc.)? |
| 7. Do you have to restrict or give up social activities (such as visiting friends, physical activity, theatre, church etc.)? |
| 8. Do your incontinence symptoms disturb your sex life? |
| 9. Does incontinence cause irritation of your external genital organs?  Do  es incontinence cause irritation of your external genital organs? |
| 10. How often must you use a protective nappy or pad? |
